# Blood Lead Levels in Asymptomatic Opium Addict Patients; a Case Control Study

**Published:** 2017-06-15

**Authors:** Kazem Ghaemi, Atefeh Ghoreishi, Navid Rabiee, Samira Alinejad, Esmaeil Farzaneh, Alireza Amirabadi Zadeh, Mohammad Abdollahi, Omid Mehrpour

**Affiliations:** 1 Atherosclerosis and Coronary Artery Research Center, Birjand University of Medical Sciences, Birjand, Iran.; 2 Department of Neurosurgery, Birjand University of Medical Sciences, Birjand, Iran.; 3 Medical Toxicology and Drug Abuse Research Center (MTDRC), Birjand University of Medical Sciences, Birjand, Iran.; 4 Department of Internal Medicine, Ardabil University of Medical Sciences, Ardabil, Iran.; 5 Toxicology and Diseases Group, Pharmaceutical Sciences Research Center, Tehran University of Medical Sciences, Tehran, Iran.

**Keywords:** Lead, substance abuse treatment centers, methadone, opium, heroin, case-control studies

## Abstract

**Introduction:**

One of the newest non-occupational sources of lead contamination is drug addiction, which has recently been addressed as a major source of lead poisoning in some countries. The present study aimed to investigate the blood lead level (BLL) of asymptomatic opium addicts.

**Methods:**

This case-control study was conducted during a one-year period to compare BLL of three groups consisting of opium addicts, patients under methadone maintenance therapy (MMT), and healthy individuals.

**Results:**

99 participants with the mean age of 55.43±12.83 years were studied in three groups of 33 cases (53.5% male). The mean lead level in opium addicts, MMT and control groups were 80.30 ± 6.03 μg/L, 67.94 ± 4.42 μg/L, and 57.30±4.77 μg/L, respectively (p=0.008). There was no significant difference in BLL between MMT and healthy individuals (p=0.433) and also between opium addicts and MMT individuals (p=0.271).Oral opium abusers had significantly higher lead levels (p = 0.036). There was a significant correlation between BLL and duration of drug abuse in opium addict cases (r=0.398, p=0.022). The odds ratio of having BLL ≥ 100 in oral opium users was 2.1 (95% CI: 0.92 - 4.61; p = 0.43).

**Conclusion:**

Based on the result of present study, when compared to healthy individuals, opium addicts, especially those who took substance orally had significantly higher levels of blood lead, and their odds of having BLL ≥ 100 was two times. Therefore, screening for BLL in opium addicts, particularly those with non-specific complaints, could be useful.

## Introduction

Lead is a versatile metal, used in approximately 900 industries (1). Due to its prevalence in various chemical derivatives (2), lead poisoning is a common problem particularly in developing countries. Lead, is easily absorbed through skin, respiratory system, and gastrointestinal tract. It readily crosses the blood-brain barrier and placenta after entering plasma and is spread in all soft and hard tissues. Abdominal pain, anemia, fatigue, joint pain, headache, memory impairment, ataxia, peripheral neuropathy, deafness, kidney disease, weakened immunity, reduced birth weight, abortion, and premature birth are some of the most important signs of lead poisoning (2-4). 

Although lead contamination usually occurs only when the individual has a history of contact with traditional sources of lead, new types of non-occupational poisoning have created some problems (5). One of the newest non-occupational sources of lead contamination is drug addiction, which has recently been addressed as a major source of lead poisoning in some countries, including Iran (6).

Inorganic lead toxicity related to intravenous injection or smoking of contaminated heroin has been reported since 1989 (7). Other instances include processed cannabis, methamphetamine, and Indian traditional herbal medicine (8-10). In Asia, opium and cannabis have been reported as the drugs abused most frequently in recent decades (11). Illicit opium may be adulterated with various materials like strychnine, paracetamol, and heavy metals such as lead and thallium (12). Salesmen and smugglers may add heavy metals to opium to raise its weight for more benefit (13, 14). Addition of lead into opium can cause an important health problem in Iran and growing reports of lead poisoning due to drug abuse are alarming. Based on above mentioned points, the present study was conducted to investigate blood lead level (BLL) in opium addicts and compare it with healthy individuals and individuals under methadone maintenance therapy (MMT). 

## Methods

***Study design and setting***

This case-control study was conducted during June to December 2014 to compare the BLL of three groups, consisting of opium addicts (hospitalized in Vali-e-Asr Hospital, Birjand, Iran), MMT cases (from the MMT clinic of Imam Reza Hospital, Mashhad, Iran), and healthy companions of the patients as control group. The participants were enrolled only if they provided direct informed consent to participate in the study. The study protocol was reviewed and approved by ethics review committee of the Birjand University of Medical Sciences under the code number 631. Researchers adhered to confidentiality of patients’ information and declarations of Helsinki.

***Participants***

Opium addicts were selected randomly from asymptomatic patients hospitalized in ophthalmology ward with other common diagnoses like cataract, glaucoma, etc. and patients under MMT were selected randomly from patients of the MMT clinic. Control group consisted of patients’ non-addict family members in order to reduce the selection bias. Case and control groups were matched based on age, sex and region. Opium addicts were enrolled in this study only if they fulfilled DSM IV criteria for substance dependence. Inclusion criterion for patients under MMT was its duration of more than six months. Control group was selected from individuals who had no history of opioid exposure. Suspected passive smoking (especially in control group), history of lead poisoning, known occupational contact with lead (e.g., plumbing, pottery, solder, battery making, and painting) and presence of an underlying systemic disease were considered as exclusion criteria. There was not any sex and age limitation.

***Data collection***

A checklist consisting of age, sex, type and duration of drug abuse, route of administration, duration of MMT, as well as BLL was developed and filled for the participants.

Two mL of venous blood was collected from all participants in complete blood count (CBC) vials, stored at 4°C and transferred to the Toxicology Laboratory of Imam Reza Hospital, Mashhad, Iran, in ice-containing flasks for measurement of lead level. BLL was measured by flame atomic absorption spectrophotometry (FAAS) method (Perkin Elmer 3030, USA). Results were expressed in μg/L. 3 trained medical students were responsible for data gathering. Laboratory technician was blind to the patients’ information. 

***Statistical analysis***

Sample size was determined based on study of Khatibi. et al. (6) considering ɑ=0.05, Ɓ=0.1 (power:0.9) and d= 1.72 (n=33 for each group). Statistical analysis was performed using SPSS software 19. Findings were reported as mean ± standard deviation or standard error and frequency (percentage). Student *t* test for parametric and Man Whitney U-test for nonparametric variables were used for two group comparison. Since BLL had a normal distribution in each group, inter-group comparison was done by a parametric test (one way ANOVA). The U.S. Department of Health and Human Services recommends that BLLs among all adults be reduced to <100 (15). Therefor 100 µg/L was considered as a cut point for determining at risk individuals. P-value less than 0.05 was considered statistically significant.

## Results

***Baseline characteristics***

99 participants with the mean age of 55.43±12.83 years (25-86) were studied in three groups of 33 cases (53.5% male). There was no significant age (p = 0.13) and sex (p = 0.07) difference between the groups. 

The route of opium administration in addict patients was ingestion in 16 (48.4%) cases, inhalation in 8 (24.2%), and mixed in 9 (27.3%). 15 (45.5%) cases used opium, 14 (42.4%) cases opium residue (Shireh), 2 (6.0%) cases heroin, and 2 (6.0%) Iranian Crystal (Heroin base). Duration of drug abuse was 18.21 ± 2.47 (1 - 50) years among the opium addicted.

Duration of MMT was 21.39 ± 3.13 (0.5- 60) months in MMT group and they had previous drug abuse duration of 10.88 ± 1.76 years. 

***Lead Levels***

The mean lead level in opium addicts, MMT and control groups were 80.30 ± 6.03 μg/L, 67.94 ± 4.42 μg/L, and 57.30±4.77 μg/L, respectively (p=0.008). There was no significant difference in BLL between MMT and healthy individuals (p=0.433) and also between opium addicts and MMT individuals (p=0.271). Table 1 compares the blood lead levels of opium addicts based on sex, age, route of administration and type of drug. Oral opium abusers had significantly higher lead levels (p = 0.036). There was a significant correlation between BLL and duration of drug abuse in opium addict cases (r=0.398, p=0.022). 

Table 2 shows the distribution of BLL below and over 100 μg/L in the studied groups. The odds ratio of having BLL ≥ 100 in oral opium users was 2.1 (95% CI: 0.92 - 4.61; p = 0.43).

## Discussion

Based on the result of present study, when compared to healthy individuals, opium addicts, especially those who took substance orally had significantly higher levels of blood lead, and their odds of having BLL ≥ 100 was two times. MMT group also had a higher BLL in comparison with healthy individuals but the difference was not significant.

**Table 1:** Blood lead levels of opium addicts based on sex, age, route of administration and type of drug

**Variable**	**N (%)**	**Lead level (μg/L)**	**P value**
**Sex**			
Male	36	83.75±5.43	0.01
Female	27	64.00±4.80	
**Age (year)**			
25-40	15	61.73±7.67	0.63
40-65	54	69.20±4.21	
>65	30	70.67±5.62	
**Route of administration**			
Ingestion	16 (48.48)	96.94 ± 8.56	0.036
Inhalation	8 (24.24)	65.63 ± 12.36	
Mix	9 (24.27)	66.56 ± 8.58	
**Type of drug**			
Heroin	2 (6.06)	49.50 ± 1.49	0.607
Opium	15 (45.45)	79.67± 8.60	
Opium residue (Shireh)	14 (42.42)	85.57± 10.08	
Iranian Crystal (Heroin based)	2 (6.06)	108.00 ± 9.37	

Data were presented as mean ± standard error.

**Table 2:** Distribution of BLL ≥ 100 μg/L between the studied groups

**Groups**	**N (%)**	**BLL < 100 **	**N (%)**	**BLL ≥ 100**	**P value**
**Opium addicts**	23 (9.7)	62.61 ± 4.63	10 (30.3)	121.00 ± 6.16	0.047
**Methadone users**	29 (87.9)	60.76 ± 3.09	4 (12.1)	120.00 ± 6.33	
**Healthy Controls**	30 (90.9)	51.40 ± 3.70	3 (9.1)	116.33 ± 9.86	

Data were presented as mean ± standard error.

Iran’s shared border with Afghanistan (the world’s largest drug manufacturer) has turned it into one of the main routes of trafficking drugs into Europe. Therefore, Iran could potentially be influenced by complications caused by drug abuse (16).

Traditional opioids (opium and its extract) were the most frequently used drugs by the opium addicts in our study, which is consistent with other studies (17-19). 9.1% of healthy individuals had BLL over 100 μg/L, which is considered acceptable for Birjand city region in comparison with the result of a study conducted on healthy individual in Arak, Iran. They reported 40.5% of participants, had BLL more than 10 μg /dl (20). U.S. Department of Health and Human Services has designated 100 µg/L of whole blood as the reference BLL for adults (15). Thus, the most recent guidelines for the management of lead-exposed adults carried out by the medical community at the current center of disease control (CDC/NIOSH) reference BLL of 100 µg/L (3).

Salehi et al. demonstrated that 40% of the studied opium addicts had a BLL over 250 μg/L (21). In addition, in a study by Shiri et al. three inpatients with lead poisoning symptoms had a mean BLL of 820 μg/L (22). The disagreement of the findings between other studies and the present study could be due to the particular method used for BLL measurement. In the cited studies, BLL was measured in symptomatic patients with confirmed diagnosis of lead poisoning, while only five of 33 (less than 5%) hospitalized patients in the present study had some degree of abdominal pain, and none of them were diagnosed as poisoned with lead.

In this study, mean BLL in opium addicts was significantly higher than in controls, which is consistent with results described by Salehi et al., Farzin et al., Abbasi et al. and Khatibi-Moghadam et al. (6,21,23-24). Increased BLL in the opium addicts could be attributed to lead contamination due to opiate abuse. The mean BLL measured in opium addicts ingesting the drug was significantly higher than other route of administration. These findings are in line with the results of Hashemi Domeneh et al. who found a significantly higher level of lead in drug abusers who prefer the oral route of ingestion (25). Previous studies demonstrated that the heat associated with smoking opium may affect the amount of lead absorbed into the blood. On the contrary, the lead is not affected in oral routes of ingestion, leading to higher levels of absorption into the blood, leading to higher BLLs (25).

BLL was higher in MMT group in comparison to healthy controls which was predictable because patients under MMT were used to opium abuse and therefore, had lead exposure, which has a long half-life in the body reported to be up to 20 years. Besides, our findings demonstrated a lower BLL in patients under MMT than opium addicts, which could represent a decreased risk of lead poisoning after withdrawal, however, the difference in BLL between the patients under methadone therapy and the opium addicts was not significant. 

Currently, opium addiction has become a major problem in some countries and has increased the rate of lead poisoning that is almost hidden for the physicians and they do not have enough training on how to treat those cases. Warning the health professionals about toxicological aspects of this issue is very important.

Therefore, screening for BLL in opium addicts, particularly those with non-specific complaints, could be useful. This is the first study in this regard, describing this phenomenon in the South Khorasan Province. The results presented here are initial findings and can be expanded in future studies employing larger sample sizes, which are more likely to be representative of the whole population.

***Limitations***

One of the limitations of our study was the use of matched controls. Matching was done just based on age, sex and region. In this regard, duration of drug abuse in the groups was not completely matched so conclusion regarding influence of MMT on BLL should be considered with caution. There was no evidence regarding the use (current or past) of opium, or other exposures that might contribute to elevated blood lead measurements in our control individuals. Therefore, we had to rely on their self-stated history. Moreover, due to lower socio-cultural status of opium addicts, explanation of the simplest objectives of the study was quite difficult. Besides, we have just evaluated BLL in opium addicts while other contaminations could be present in opium that need further study. Another limitation of our study was lack of confirmation of absence of lead contamination of blood by other sources. However, a history of lead poisoning and known occupational contact with lead (e.g., plumbing, pottery, solder, battery making, and painting) were considered as the exclusion criteria. Moreover, it is possible that the elevated BLLs reported in this study were the result of patient exposure to a wide range of potential lead sources that were impossible to control for such as food, air pollution to lead, etc. Unfortunately, opium used by addicts was not accessible and we couldn’t analyze them for lead to ascertain the conclusion. 

## Conclusion:

Based on the result of present study, when compared to healthy individuals, opium addicts, especially those who took substance orally had significantly higher levels of blood lead, and their odds of having BLL ≥ 100 was two times. Therefore, screening for BLL in opium addicts, particularly those with non-specific complaints, could be useful. 
